# A possible home for a bizarre Carboniferous animal: is *Typhloesus* a pelagic gastropod?

**DOI:** 10.1098/rsbl.2022.0179

**Published:** 2022-09-21

**Authors:** Simon Conway Morris, Jean-Bernard Caron

**Affiliations:** ^1^ Department of Earth Sciences, University of Cambridge, Downing Street, Cambridge CB2 3EQ, UK; ^2^ Department of Natural History, Royal Ontario Museum, 100 Queen's Park, Toronto, Ontario, Canada M5S 2C6; ^3^ Department of Ecology and Evolutionary Biology, University of Toronto, 25 Willcocks Street, Toronto, Ontario, Canada M5S 3B2; ^4^ Department of Earth Sciences, University of Toronto, 22 Russell Street, Toronto, Ontario, Canada M5S 3B1

**Keywords:** *Typhloesus*, Carboniferous, molluscs

## Abstract

By contrast to many previously enigmatic Palaeozoic fossils, the Carboniferous metazoan *Typhloesus* has defied phylogenetic placement. Here, we document new features, including possible phosphatized muscle tissues and a hitherto unrecognized feeding apparatus with two sets of *ca* 20 spinose teeth whose closest similarities appear to lie with the molluscan radula. The ribbon-like structure, located well behind the mouth area and deep into the anterior part of the body, is interpreted as being in an inverted proboscis configuration. Gut contents, mostly conodonts, in the midgut area demonstrate that *Typhloesus* was an active predator. This animal was capable of propelling itself in the water column using its flexible body and a prominent posterior fin. The affinity of *Typhloesus* as a pelagic mollusc remains problematic but may lie more closely with the gastropods. Heteropod gastropods share with *Typhloesus* an active predatory lifestyle and have a comparable general body organization, albeit they possess characteristic aragonitic shells and their origins in the Jurassic post-date *Typhloesus*. *Typhloesus* may represent an independent radiation of Mid-Palaeozoic pelagic gastropods.

## 1. Introduction

Recent years have witnessed a steady thinning in the ranks of ‘weird wonders’, that is taxa with unfamiliar, if not bizarre, body forms that seemingly preclude confident assignment to known groups. Albeit with varying degrees of confidence, many such taxa have now been shunted to reasonably secure phylogenetic destinations (e.g. [1,2]). The majority of these test cases have come from Cambrian Fossil-Lagerstätten, notably the Burgess Shale (and geographically adjacent equivalents) and Chengjiang, but younger Lagerstätten still house a number of evolutionary enigmas. Among these is the Upper Mississippian (*ca* 330 Myr) Bear Gulch Limestone [3], home to the bizarre *Typhloesus wellsi* that half-jocularly was referred to as an ‘alien goldfish’ [4].

Originally hailed as the long-sought-after conodont animal [5], it transpired that the conodonts were ingested. Dubbed the ’conodontochordates’ and despite being vaguely fish-like, their highly unusual anatomy not only ruled out comparison with the chordates but any other phylum [6]. Since then, with the exception of a detailed description of conodont apparatuses [7] and unpublished work on its taphonomy [8], *Typhloesus* has received only passing mention (e.g. [3,9,10]). Here, we report new information on this animal, especially that pertaining to the feeding apparatus. This organ is consistent with the predatory habits of *Typhloesus*, but it also suggests that despite its unusual appearance this animal may be a pelagic mollusc.

## 2. Methods

**Figure 1.**
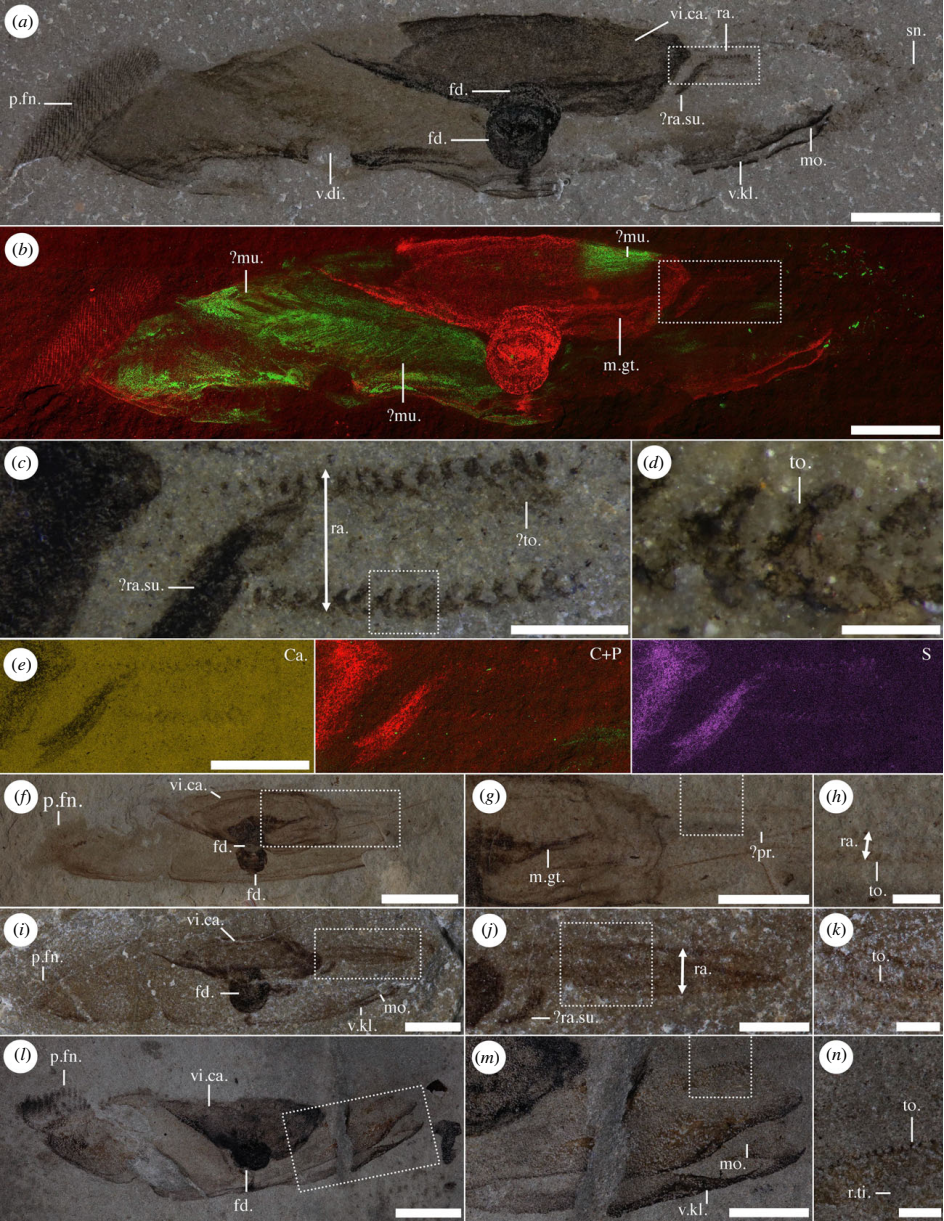
*Typhloesus wellsi* showing radula. (*a*–*e*) ROMIP 48528; (*a*) full view; (*b*) elemental map showing possible phosphatized traces of muscles tissues (carbon in red, phosphorus in green); (*c*) close-up of the radula; (*d*) details of radular teeth; (*e*) elemental maps of the radula showing slight enrichment in carbon (centre—red)) and sulfur (right) but little evidence of calcium (left) and phosphorus (centre—green). (*f*–*h*) ROMIP 48526; (*f*) full view; (*g*) close-up of the radula; (*h*) details of radular teeth. (*i*–*k*) ROMIP 47470; (*i*) full view; (*j*) close-up of the radula; (*k*) details of radular teeth. (*l*–*n*) ROMIP 58284; (*l*) full view; (*m*) close-up of the radula; (*m*) details of radular teeth. All specimens flipped horizontally, anterior to the right. fd., ferrodiscus; m.gt., midgut; mo., mouth; ?mu., possible musculature; p.fn. posterior fin; ra., radula; ?pr., proboscis; ?ra.su., radula support; r.ti., reticulate tissues; sn., snout; to., tooth; v.di., ventral diastema; v.kl., ventral keel; vi.ca., visceral capsule. Scale bars = 10 mm (*f*,*l*); 5 mm (*a*,*b*,*g*,*i*,*m*); 2 mm (*e*,*j*); 1 mm (*c*,*h*,*k*,*n*); 0.25 mm (*d*).

Over a number of years, by donation and purchase, the Royal Ontario Museum acquired an important collection of *Typhloesus*. Several specimens were mentioned by Conway Morris [6], but with the exception of one specimen [7] none of the remaining specimens have been described. The entire collection was initially studied under polarized light with a binocular microscope. Two specimens (ROMIP 48526 and 48528) were examined with a scanning electron microscope (FEI Quanta 200 FEG) at the University of Windsor Great Lakes Institute for Environmental Research, Canada. Elemental mapping (figure 1*b*,*e*; electronic supplementary material, figures S1*a*–*h* and S2*g*–*j*) was performed with energy dispersive spectroscopy (EDS) using an EDAX Octane Plus Silicon Drift X-Ray detector with a 12 kV beam accelerating voltage under 70 Pa chamber pressure (low vacuum).

## 3. Description

For the most part examination of the Royal Ontario Museum material confirms earlier descriptions [5,6]. A key discovery, however, is the recognition of a radula-like organ in the region already identified as the foregut (figure 1). Specifically, on the ventral margin this consists of two rows of *ca* 20 posteriorly recurved teeth (*ca* 200 µm high) separated by *ca* 1 mm (figure 1*c*,*d*; electronic supplementary material, figure S2*a*–*e*). The length of the entire structure is about 4 mm. The teeth have a triangular shape with the wider base at the front (electronic supplementary material, figure S2*d*,*e*). The smallest teeth are at the posterior end and are more spaced from each other (electronic supplementary material, figure S2*c*); the posterior three teeth are not aligned with the other teeth, but seem to be positioned along a curve (electronic supplementary material, figure S2*c*). Other specimens appear to show one (figure 1*n*) or two rows (figure 1*h*,*j*,*k*) with alternating insertions, but with a narrower distance between the rows. We interpret this variation as the result of the rotation of either the entire specimen relative to the bedding plane or the radula itself within the body cavity during decay. As it happens, this dentition had been documented earlier as ‘a series of block-like structures’ [6, p. 607], but at the time was tentatively identified as musculature. There are also variably preserved traces of adjacent tissue. One specimen shows elongate structures adjacent to the anterior end of one of the radular rows (figure 1*c*; electronic supplementary material, figure S2*c*). Their identity remains uncertain, but conceivably they represent ancillary teeth. Another specimen shows that the base of the radula has a reticulate and fibrous texture (figure 1*n*).

In addition to the recognition of a radula-like organ, our restudy has also led to a number of new observations. Elemental analysis shows the presence of carbon, phosphorus and sulfur in discrete parts of the specimens, and reveals new features of anatomy. In particular, in ROMIP 48528 (figure 1*b*; electronic supplementary material, figure S1*c*) phosphorus in the posterior section picks out broad blocks of tissue that seem to form two sets inclined in opposite directions. Most likely these represent a propulsive musculature. Previously identified cuticular fibres ([6], figs. 56, 57, pl. 7, figs. 61, 62) in the same region have approximately the same orientation, but an equivalence to these blocks is uncertain. In passing, if the tentative identification of longitudinal muscles in this area ([6], fig. 58, pl. 7, fig. 66) is correct then these clearly lie at a different level to the larger blocks and may have been more surficial. Towards the anterior of the midgut the dorsal side shows another fibrous area, again possibly a musculature (figure 1*b*; electronic supplementary material, figure S1*g*). In ROMIP 48526 phosphorus has a more extensive distribution, but other regions of the midgut also have a fibrous appearance (electronic supplementary material, figure S2*i*). Alternate contractions of such muscles may have helped to dilate the midgut during feeding as well as subsequently expelling digested material. In the earlier description, the midgut was assumed to be a voluminous organ. In ROMIP 48526, however, the anterior region of the midgut is associated with a narrower strand that conceivably represents an extension of the foregut (figure 1*g*; electronic supplementary material, figure S2*f*–*j*) (see also below). Somewhat similar structures in other specimens ([6], pl. 2, figs. 12, 19; pl. 4, figs. 32, 35, 36, 37, pl. 5, figs. 38, 39, 40, 41) have been interpreted as part of the blood vascular system but are possibly also part of the midgut. A large central fusoid area within the body, possibly bilaterally organized, encapsulates the midgut and part of the foregut (figure 1*a*,*f*,*i*,*l*; electronic supplementary material, figure S2*f*). This structure is wider at the front and tapers towards the rear. Previously interpreted as a foregut itself, we re-interpret this structure as equivalent to a visceral capsule. Notably this structure is preserved (and sometime the only structure to remain visible) in all specimens studied, and shows an enrichment in carbon, sulfur and to a lesser extent phosphorus, but does not include calcium (figure 1*b*; electronic supplementary material, figures S1*e*,*f*,*h* and S2*i*). Phosphorus is likely associated with surficial muscles, but carbon and sulfur might represent more refractory and tougher tissues. The ventral keel and ferrodiscus are also preserved in a similar manner, albeit with higher concentration of the above elements (figure 1*b*; electronic supplementary material, figures S1*e*,*f* and S2*h*,*i*).

In the earlier description [6], a pair of prominent keels were identified on the ventral side, and inferred to diverge adjacent to a pre-oral area. ROMIP 58284 supports this reconstruction (figure 1*l*,*m*), as well as other specimens (figure 1*a*,*i*), but suggests the pre-oral area was at least as extensive as previously depicted and may have been an important ancillary in prey capture.

The earlier interpretation [6] of *Typhloesus* also suggested the gut was blind and the ROMIP material provides no firm evidence for any posterior extension from the midgut. ROMIP 48526 shows clear gut contents within the midgut area. Finally, we draw attention to examples of a conspicuously well-preserved tail that in addition to the fin-rays (rods and synapticulae) displays the fin bars (figure 1*a*,*l*).

## 4. Taphonomy

Preservation of *Typhloesus* was discussed by Conway Morris [6]. Preliminary observations using EDAX indicated a number of elements, but significantly no iron associated with the so-called ferrodiscus. Here, our more comprehensive elemental mapping unsurprisingly records carbon. The association of phosphorus with possible musculature was reviewed above. Sulfur—together with carbon—is also widely distributed but occur in discrete parts of the body or organs. Sulfur may reflect finely disseminated pyrite (or its weathered equivalents) and suggest diagenetic sulfurization reminiscent to the preservation of conodont eyes in some Silurian deposits [11].

## 5. Discussion

**Figure 2.**
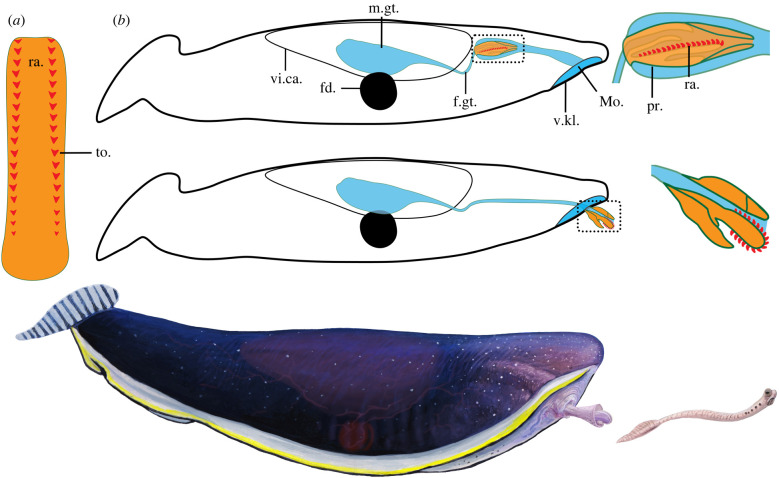
*Typhloesus wellsi*: anatomical schematic diagrams and artistic reconstruction. (*a*) Interpretative reconstruction of the radula fully outstretched as seen from above, anterior to the top, showing two main rows of lateral teeth (red triangles) decreasing in size towards the rear; (*b*) interpretative sagittal sections of the body showing the gut system (blue) with a blind gut and the proboscis with the radula complex (orange) in a fully inverted (top) and everted (bottom) position. Framed areas, close-ups of anterior region of the proboscis; (*c*) artistic representation of *Typhloesus wellsi* in the process of catching its conodont prey using its everted proboscis and radula. Drawing by Joschua Knüppe © Royal Ontario Museum. fd., ferrodiscus; m.gt., midgut; mo., mouth; ra., radula; pr., proboscis; vi.ca., visceral capsule.

Identification of the toothed feeding apparatus throws further light on the functional anatomy of *Typhloesus*, and potentially its wider relationships. The location of the teeth in the posterior section of the foregut, as well as their direction of curvature, suggests that to function effectively most of the foregut would have had to evert in order to bring the teeth into a position to seize prey (figure 2). The alternative that prey was swallowed and only then engaged in trituration seems less likely given none of the teeth are molariform. On the former supposition, eversion of the foregut would most likely have been achieved by a hydrostatic mechanism whereby the foregut was enclosed in a fluid-filled body cavity. Such an arrangement finds counterparts in groups such as the gastropods (notably the so-called acrembolic-type proboscis as against the more usual simple retraction) [12] and the rhynchocoel of nemerteans [13]. Hydrostatic mechanisms in animals usually depend on muscular contraction, including the employment of retractors. In *Typhloesus* a paired structure close to the foregut–midgut boundary (figure 1*a*,*c*,*j*; electronic supplementary material, figure S1*e*,*f*) was tentatively identified as part of a vascular system (the ‘valvaforis’ of Conway Morris [6]). Its paired arrangement suggests that it is unlikely to be something akin to the radula sac of molluscs. It appears to have a carbonaceous composition (with additional sulfur), but unlike putative musculature no enrichment in phosphorus (electronic supplementary material, figure S1*g*). It remains possible that this tissue was originally muscular and involved with retraction of the adjacent foregut, but perhaps a more likely possibility is as bolsters to support the radula complex. Whilst hypothetical it is also conceivable that in life eversion of the foregut was forcible and rapid [14].

We suggest that the radula arrangement seen in *Typhloesus* is most reminiscent of the molluscs. We are mindful that jaws are rampantly convergent (e.g. [15]) and radular-like structures occur elsewhere, such as in the amphinomid polychaete *Chloeia* [16]. In no other respect, however, is *Typhloesus* annelid-like, not least in the absence of chaetae or obvious metamerism.

Accepting that the rows of teeth are equivalent to the ribbon-like radula does not in itself assist in assigning *Typhloesus* to a particular group of molluscs. Although classically the radula is conceptualized as a polydentate ribbon, more generally along with associated jaws [17] the diversity of radular arrangements is immense, sometimes even in relatively small taxonomic groups [18,19]. In molluscs such as the cone shells it can show a dramatic reduction reflecting specialized ways of life (e.g. [20,21]). In general, however, the canonical radula consists of a central rachidian tooth, flanked by lateral teeth. In the aplacophorans, however, the median tooth is absent and the distichous arrangement in some taxa [22] is reminiscent of *Typhloesus*. The vermiform aplacophorans are highly derived molluscs and otherwise have no obvious similarities (e.g. spicules) to *Typhloesus*.

Aplacophorans also are wholly benthic. The apparent disparity between the anatomy of *Typhloesus* and other pelagic molluscs makes further phylogenetic assessment hazardous. In the earlier description [6] passing comparison was made to extant pelagic gastropods [23], notably a degree of similarity between the posterior fin of *Typhloesus* and the fin-like foot of heteropod taxa such as *Carinaria* and *Pterosoma* (e.g. [24]). In nearly all respects, however, a direct comparison with the heteropods is tenuous. Nor would it be easy to reconcile with their fossil record, which is based entirely on the shells. Heteropod-like gastropods may date back to the Triassic [25], underwent a rapid radiation in the early Jurassic, perhaps as a response to widespread bottom-water anoxia [26]. The five clades identified in the early Mesozoic have no obvious phylogenetic link to the younger heteropods that gave rise to the extant taxa [27]. The origins of the modern taxa date from the Cretaceous and occur in the form of the Atlantidae [28]. The more derived carinariids (and pterotracheiids) [29], to which *Typhloesus* might bear some closer comparison, only appeared in the Cainozoic. Although the fossil record of the heteropods is patchy, their stratigraphic distribution is broadly consistent with molecular data. If, therefore, *Typhloesus* is a holopelagic gastropod it would be convergent on a carinariid-like form, representing a Palaeozoic migration into the pelagic zone.

There is an additional line of evidence that potentially might help resolve the phylogenetic position of *Typhloesus*. This entails a general characteristic of gastropods that occurs early in ontogeny and is referred to as torsion. At least partial detortion, however, is also known, notably in some opisthobranchs [30], including the pelagic nudibranchs [31]. It also needs to be acknowledged that among extant heteropods, especially the shell-less pterotracheiids, the elongate body displays an oesophagus that extends in linear fashion to the visceral nucleus [23], so that even shortly after larval metamorphosis [32] torsion is far from self-evident. Given the size range of *Typhloesus* [6], with no obvious juveniles (let alone earlier larval stages), direct identification of torsion might be difficult and is further compounded by alternative interpretations of the soft-part anatomy. One notable feature of *Typhloesus* is the blind gut, an observation supported by the absence of any gastric contents in the posterior section of the body. However, in one specimen (ROMIP 48526, figure 1*f*,*g*, electronic supplementary material, figure S1*g*) tissue identified as the midgut could, given the degree of compression, be reinterpreted as a hindgut leading to an anus. In such a scenario this area would be equivalent to the visceral nucleus with a head–foot complex to the anterior and in the opposite direction the elongate tail. Some other specimens may show comparable features, but if so remain much less well-defined. We conclude that a place for *Typhloesus* among the gastropods is plausible, but acknowledge that similarities to the molluscs, let alone the heteropods, may be the result of convergence [33].

## 6. Conclusion

The Carboniferous animal *Typhloesus* possesses a radula-like structure, suggesting its phylogenetic position may be resolved as a mollusc and analogous to the extant pelagic heteropods, but more precise pronouncements of its relationships are hampered by the unique aspects of its morphology (notably the so-called ferrodiscus) and the sparsity of equivalent soft-bodied fossils in Palaeozoic deposits.

## Data Availability

All fossil material in this study are reposited in the Invertebrate Palaeontology collections at the Royal Ontario Museum. The data are provided in the electronic supplementary material [34].

## References

[1] Caron J-B, Cheung B. 2019 *Amiskwia* is a large gnathiferan with complex gnathostomulid-like jaws. Commun. Biol. **2**, e164. (10.1038/s42003-019-0388-4) PMC6499802 31069273

[2] Zhao Y, Vinther J, Parry LA, Wei F, Green E, Pisani D, Hou X, Edgecombe GD, Cong P. 2019 Cambrian sessile, suspension feeding stem-group ctenophores and evolution of the comb jelly body plan. Curr. Biol. **29**, 1112-1125.e2. (10.1016/j.cub.2019.02.036) 30905603

[3] Hagadorn JW. 2002 Bear Gulch: an exceptional upper Carboniferous Plattenkalk. In Exceptional fossil preservation: a unique view on the evolution of marine life (eds DJ Bottjer et al.), pp. 167-183. New York, NY: Columbia University Press.

[4] Conway Morris S. 2005 Aliens like us? Astron. Geophys. **46**, 4.24-4.26.

[5] Melton W, Scott HW. 1973 Conodont-bearing animals from the Bear Gulch Limestone, Montana. Spec. Pap. Geol. Soc. Am. **141**, 31-65.

[6] Conway Morris S. 1990 *Typhloesus wellsi* (Melton and Scott, 1973), a bizarre metazoan from the Carboniferous of Montana, U.S.A. Phil. Trans. R. Soc. Lond. B **327**, 595-624. (10.1098/rstb.1990.0102)

[7] Purnell MA. 1993 The *Kladognathus* apparatus (Conodonta, Carboniferous): homologies with ozarkodinids, and the prioniodinid Bauplan. J. Paleont. **67**, 875-882. (10.1017/S0022336000037136)

[8] Thomas N. 2004 The taphonomy of a Carboniferous Lagerstätte: the invertebrates of the Bear Gulch Limestone. PhD thesis, University of Leicester, Leicester.

[9] Moore RA, McKenzie SC, Lieberman BS. 2007 A Carboniferous synziphosurine (Xiphosaura) from the Bear Gulch Limestone, Montana, USA. Palaeontology **50**, 1013-1019. (10.1111/j.1475-4983.2007.00685.x)

[10] Donoghue PCJ, Purnell MA. 2009 Distinguishing heat from light in debate over controversial fossils. Bioessays **31**, 178-189. (10.1002/bies.200800128) 19204990

[11] von Bitter PH, Purnell MA, Tetreault DK, Stott CA. 2007 Eramosa Lagerstätte—exceptionally preserved soft-bodied biotas with shallow-marine shelly and bioturbating organisms (Silurian, Ontario, Canada). Geology **35**, 879-882. (10.1130/G23894A.1)

[12] Golding RE, Ponder WF, Byrne M. 2009 The evolutionary and biomechanical implications of snout and proboscis morphology in Caenogastropoda (Mollusca: Gastropoda). J. Nat. Hist. **43**, 2723-2763. (10.1080/00222930903219954)

[13] Chernyshev AV. 2015 CLSM analysis of the phalloidin-stained muscle system of the nemertean proboscis and rhynchocoel. Zool. Sci. **32**, 547-560. (10.2108/zs140267) 26654037

[14] Schulze JR, Jan I, Sangha G, Azizi E. 2019 The high speed radular prey strike of a fish-hunting cone snail. Curr. Biol. **29**, R788-R789. (10.1016/j.cub.2019.07.034) 31430472

[15] Hochberg R, Wallace RL, Walsh EJ. 2015 Soft bodies, hard jaws: an introduction to the symposium, with rotifers and models of jaw diversity. Integr. Comp. Biol. **55**, 179-192. (10.1093/icb/icv002) 25796591 PMC6296403

[16] Dales RP. 1962 The polychaete stomodaeum and the inter-relationships of the families of Polychaeta. Proc. Zool. Soc. Lond. **139**, 389-428. (10.1111/j.1469-7998.1962.tb01837.x)

[17] Lobo-da-Cunha A. 2019 Structure and function of the digestive system in molluscs. Cell Tissue Res. **377**, 475-503. (10.1007/s00441-019-03085-9) 31478138

[18] Gosliner TM. 1994 Gastropoda: Opisthobranchia. In Microscopic anatomy of invertebrates (eds FW Harrison, AJ Kohn), vol. 5: Mollusca I, pp. 253-355. New York, NY: Wiley-Liss.

[19] Kantor YI, Taylor JD. 2002 Foregut anatomy and relationships of raphitomine gastropods (Gastropoda: Conoidea: Raphitominae). Boll. Malacol. Suppl. **4**, 83-110.

[20] Kantor YI, Taylor JD. 2000 Formation of marginal radular teeth in Conoidea (Neogastropoda) and the evolution of the hypodermic envenomation mechanism. J. Zool. **252**, 251-261. (10.1111/j.1469-7998.2000.tb00620.x)

[21] Vortsepneva E, Tzetlin A, Kantor Y. 2019 First ultrastructural study of the formation of the hypodermic radula teeth of *Conus* (Neogastropoda: Conidae). J. Molluscan Stud. **85**, 184-196. (10.1093/mollus/eyz010)

[22] Scheltema AH. 2014 The original molluscan radula and progenesis in Aplacophora revisited. J. Nat. Hist. **48**, 2855-2869. (10.1080/00222933.2014.959573)

[23] Lalli CM, Gilmer RW. 1989 Pelagic snails: the biology of holoplanktonic gastropod mollusks. Stanford, CA: Stanford University Press.

[24] Tesch JJ. 1949 Heteropoda. Dana Rep. **34**, 1-53.

[25] Pieroni V, Nützel A. 2020 *Freboldia carinii* sp. nov. from the Middle Triassic Brembana valley (Esino Limestone, Southern Alps, Italy)—possibly the oldest known holoplanktonic gastropod. Neues Jahrb. Geol. Paläont. Abh. **298**, 9-15. (10.1127/njgpa/2020/0929)

[26] Hart MB, Wall-Palmer D, Janssen AW, Smart CW. 2020 Some observations on the geological history of the holoplanktonic gastropods. Proc. Geol. Assoc. **131**, 443-449. (10.1016/j.pgeola.2020.07.009)

[27] Nützel A, Schneider S, Hülse P, Kelly SRA, Tilley L, Veit R. 2016 A new early Jurassic gastropod from Ellesmere Island, Canadian Arctic—an ancient example of holoplanktonic gastropods. Bull. Geosci. **91**, 1-14.

[28] Wall-Palmer D, Janssen AW, Goetz E, Choo LQ, Mekkes L, Katja TCA. 2020 Fossil calibrated molecular phylogeny of atlantid heteropods (Gastropoda, Pterotracheoidea). BMC Evol. Biol. **20**, e124. (10.1186/s12862-020-01682-9) PMC7507655 32957910

[29] Thiriot-Quiévreux C, Seapy RR. 1997 Chromosome studies of three families of pelagic heteropod molluscs (Atlantidae, Carinariidae, and Pterotracheidae) from Hawaiian waters. Can. J. Zool. **75**, 237-244. (10.1139/z97-030)

[30] Mikkelsen PM. 2002 Shelled opisthobranchs. Adv. Mar. Biol. **42**, 67-136. (10.1016/S0065-2881(02)42013-5) 12094725

[31] Bonar DB, Hadfield MG. 1974 Metamorphosis of the marine gastropod *Phestilla sibogae* Bergh (Nudibranchia: Aeolidacea). I. Light and electron microscopic analysis of larval and metamorphic stages. J. Exp. Mar. Biol. Ecol. **16**, 227-255. (10.1016/0022-0981(74)90027-6)

[32] Owre HB. 1964 Observations on the development of heteropod molluscs *Pterotrachea hippocampus* and *Firoloida desmaresti*. Bull. Mar. Sci. Gulf Caribb. **14**, 529-538.

[33] Conway Morris S 2015 The runes of evolution: how the universe became self-aware. Conshohocken, PA: Templeton Press.

[34] Conway Morris S, Caron J-B. 2022 A possible home for a bizarre Carboniferous animal: is *Typhloesus* a pelagic gastropod? Figshare. (10.6084/m9.figshare.c.6186132)

